# Distillation as a meaningful water source for lithic lichens: the Negev case

**DOI:** 10.1007/s00425-025-04916-6

**Published:** 2026-01-03

**Authors:** Giora J. Kidron, Rafael Kronenfeld, Abraham Starinsky

**Affiliations:** 1 Institute of Earth Sciences, The Hebrew University, Givat Ram Campus, 91904 Jerusalem, Israel; 2 Meteorological Unit, Israel Meteorological Service, Kibbutz Sede Boqer, Israel

**Keywords:** Cobbles, Dew, Indirect rain water (IRW), Lithobionts, Non-rainfall water (NRW)

## Abstract

**Main conclusion:**

In the Negev, substantial vapor stems from the wet soil following rain events and therefore cannot be considered as dew but rather as distillation. Distillation provided ~ 35% and ~ 60% of the vapor-driven liquid for the cobbles and rock slabs, respectively, implying that lithobionts may benefit from vapor condensation also in non-dewy deserts.

**Abstract:**

Lithic chlorolichens (lichens with green algae as photobionts) and cyanobacteria cover almost all rock surfaces in the Negev Highlands, where chlorolichens are believed to mainly benefit from non-rainfall water (NRW), i.e., dew and vapor at high relative humidity. Since chlorolichens may also inhabit non-dewy deserts and vapor may also stem from the wet soil (which once condenses is termed distillation), we hypothesized that vapor that stems from the wet soil may also benefit lithic chlorolichens. To evaluate the potential amount accumulated on these rocky surfaces, whether by NRW or soil vapor plus distillation (jointly termed as indirect rain water, IRW), 3-year-long measurements were conducted in the Negev using cloths attached to a pair of rock slabs and a pair of cobbles. Taking 0.05 (reflecting vapor adsorption) and 0.1 mm (reflecting vapor condensation), which allows for net photosynthesis by chlorolichens and cyanobacteria, respectively, we found that: (1) the average number of days with NRW and IRW ≥ 0.05 mm was respectively 128.7 days and 28.0 days (for cobbles) and 37.3 days and 19.3 days (for rock slabs), with dew (which occurs along the year) and distillation (limited to days after rain events) occurring respectively for 36.7 days and 20.0 days (cobbles) and 28.0 days and 6.0 days (rock slabs), (2) average annual amounts of NRW and IRW ≥ 0.05 mm were respectively 11.5 mm and 3.9 mm (for cobbles) and 2.7 mm and 1.8 mm (for rock slabs), with dew and distillation being respectively 4.7 mm and 3.1 mm (for cobbles) and 0.5 mm and 0.9 mm (for rock slabs), (3) average annual daytime duration of > 0.05 mm for NRW and IRW were respectively 307.8 h and 83.9 h (for cobbles) and 81.0 h and 46.7 h (for rock slabs) with dew and distillation lasting respectively 103.8 h and 60.2 h (for cobbles) and 10.3 h and 17.6 h (for rock slabs). Given that daylight duration primarily dictates growth, we may conclude that: (1) cobbles receive substantially higher amounts of NRW and IRW than rock slabs, (2) the amount of distillation received on cobbles (3.1 mm) was not substantially lower than that of dew (4.7 mm). As far as the annual daylight wetness duration for cobble-dwelling lichens is concerned, distillation provided 36.7% of the total duration provided by vapor. Since IRW may occur also in dewless deserts, such as the Mojave Desert, it may partially explain lithic lichen inhabitation in the Mojave and other non-dewy deserts.

## Introduction

Playing an important role in carbon fixation in deserts, lithic organisms/microorganisms attracted the attention of many scholars (Lange et al. 1970; Friedmann and Galun 1974; Nash et al. 1977; de los Rios et al. 2007; Davila et al. 2008; Garvie et al. 2008; Wierzchos et al. 2006; Azua-Bustos et al. 2012; DiRuggiero et al. 2013; McKay et al. 2024). This is especially the case with lithic crustose lichens (lichens closely attached to rock and cobble surfaces), whose abundance in deserts over other growth forms of lichens has been long ago noted (Fink 1909; Nash et al. 1977; Lisci et al. 2003; Favero-Longo et al. 2004). Commonly, these lithic organisms/microorganisms are assumed to benefit also from non-rainfall water, principally dew and fog (Smith et al. 2000; Warren-Rhodes et al. 2013; Davila et al. 2008; DiRuggiero et al. 2013; Gauslaa 2014; Bernhard et al. 2018), but yet, lithobionts and among them chlorolichens also inhabit rock surfaces and especially rock fragments (boulders, stones, cobbles) in deserts that do not benefit from non-rainfall water (NRW), whether dew or fog, such as those in the southwestern parts of the USA (Nash et al. 1977).

High cover of crustose lithobionts characterizes the Negev Desert. The high cover and diversity of crustose lithobionts in the Negev Highlands is notorious among deserts, especially hyperarid deserts (Friedmann and Galun 1974). The rock surfaces (either extensive rock outcrops or isolated rocks partially embedded in soil) and cobbles (rock fragments with a diameter of 6.3–20 cm located on the ground; Blott and Pye 2012) in particular are almost entirely covered by lithobionts (Kidron and Temina 2013), mainly chlorolichens, i.e., lichens with green algae as photobionts. Lithic cyanobacteria also cover parts of the drainage basin, principally confined to the sun-exposed, south- and east-facing bedrocks. It was widely accepted that the lush cover of lithobionts can be attributed to dew, which, according to Evenari et al. (1971), contributed about 33 mm of water, adding about 30% to the long-term rain precipitation of 95 mm that characterizes the Negev Highlands.

The confinement of cyanobacteria to the south- and east-facing bedrocks was especially odd since all cobbles at these aspects are covered with chlorolichens. The incoherence was clarified only by direct NRW measurements on the rock surfaces. By attaching thin (0.1 cm-thick) cloths of high absorbance to various rock and cobble surfaces, a clear picture has emerged. Unlike the cobbles that facilitated dew formation, the surfaces of the bedrocks were too warm to allow vapor condensation. These surfaces were also too warm to facilitate the accumulation of sufficient vapor that may allow net photosynthesis by eukaryotes (Kidron et al. 2014, 2016, 2026).

Knowledge regarding the required water thresholds for prokaryotes and eukaryotes in the Negev is mainly based on thorough research conducted by Lange and co-workers between 1969 and 1980. Whereas prokaryotes such as cyanobacteria and cyanolichens (lichens with cyanobacteria as photobionts) necessitate liquid water to initiate activity, whether respiration or net photosynthesis, eukaryotes such as green algae and chlorolichens were found capable of initiating their activity also at high relative humidity (RH). Accordingly, whereas eukaryotes may initiate respiration already at RH of 70%, they necessitate RH of 80% to initiate net photosynthesis (Kappen et al. 1979; Lange et al. 1986).

Using *Ramalina maciformis* (Delile) Bory as a model example for the Negev Desert, a comparison between the amount of water adsorbed onto the lichen and cloths was possible (Kidron et al. 2014; Kidron and Starinsky 2019). It was determined by attaching side by side cloths and sections of the lichen thalli of *R. maciformis,* both of the same dimension (~ 2.5 × 1.5 cm), to different rock surfaces and comparing the amount of water adsorbed by the lichen thalli and the cloths, as the percent of the dry mass (% of water content) or in millimeters (by dividing the amount of water by the surface area of the thalli or cloth). Corresponding to the equation *Y* = −0.0042*x*^2^ + 0.94x + 1.4 and yielding a high correlation of *r*^2^ = 0.88 (Kidron et al. 2014), the water thresholds for respiration at RH = 70% (equivalent to 14% of the dry mass of *R. maciformis* sensu; Kappen et al. 1979 and 13.7% of the cloth) and net photosynthesis at RH = 80% (equivalent to 20% of the dry mass of *R. maciformis* sensu; Kappen et al. 1979 and 18.5% of the cloth) corresponded, respectively, to 0.03 and 0.05 mm (Kidron and Starinsky 2019). *R. maciformis* was found to be also adapted to a gradual increase in its water content, which reflects high adaptation to NRW. Furthermore, as substantiated by Lange et al. (1970), crustose lichens which abound in the Negev exhibited similar threshold values to those of *R. maciformis.*

The above-mentioned amounts were substantially lower than the amount that reflects sufficient vapor accumulation, which will result in vapor condensation (Beysens 1995, 2018). Vapor condensation will take place at 0.1 mm, which is required to facilitate cell turgidity and the activity of prokaryotes (Lange et al. 1992). It was therefore substantiated that whereas liquid water (rain and dew of ≥ 0.1 mm) is required for the activation of cyanobacteria, non-rainfall water (NRW) is sufficient to activate chlorolichens (Lange et al. 1986; Phinney et al. 2019). Whereas a minimum amount of 0.03 mm will initiate respiration, a minimum amount of 0.05 mm will be required to initiate net photosynthesis. Thus, in addition to rain, NRW (whether dew, fog, or vapor at high RH) may serve as an important water source for lithobionts in the Negev.

While indicating that a few hours were needed to achieve net photosynthesis at high RH, an exact value was not provided (Lange 1969; Kappen et al. 1979). However, based on the reported condensation rate of ~ 0.025 mm h^−1^ (Kidron 2000a), one may conclude that RH of 80% will be reached after 2 h, implying net photosynthesis following ≥ 3 h.

The high adaptation of *R. maciformis* to dew may have various advantages. Thus, for instance, no meaningful supersaturation was detected even when the maximal dew water content was reached and therefore, no inhibition of photosynthesis due to supersaturation, as may sometimes be the case (Stanton et al. 2023), was detected. This was explicitly expressed by Lange (1969), who concluded that the supersaturation ‘do not occur at all or occur only to a minor extent’. As far as the temperatures are concerned, *R. maciformis* also exhibited an adaptation to the mild Negev temperatures that characterize the dewy season, thus avoiding excessive carbon loss following rain during the cold winter (Meyer et al. 2024). The optimal temperature of 20 ℃, as reported by Lange (1969), reflects the early morning temperatures in the fall dewy season of the Negev (Bitan and Rubin 1991).

Recent findings pointed, however, to the fact that vapor that stems from the wet soil may also provide water to lithobionts. As thoroughly described by Monteith (1957) and other scholars (e.g., Jacobs et al. 1990; Li et al. 2023), vapor that stems from the wet soil may condense (a process termed by Monteith 1957 as distillation) and wet the lower parts of plants. As thoroughly discussed, both dew and distillation result from radiative cooling. Soil vapor condensation may also be reflected by wet-dry cycles (WDCs) that are visibly noted after rain. Although the surface may dry out during midday, commonly 1–3 days after the rain event, it may be rewetted during the following night and morning, resulting in WDCs that may last up to a week or two following medium- and high-depth rain events (Kidron et al. 2022, 2025). This vapor transport takes place within the upper soil layer following temperature-induced vapor flux when vapor that stems from the warm and wet subsurface layer during the night is emitted towards the cool/cold surface. This vapor, whether undergoing condensation or not, may also benefit lithobionts.

WDCs were also observed in the Negev (Kidron et al. 2022, 2024, 2025), and vapor that was emitted from the soil was recently found to provide water to cobbles and rock slabs lying on the ground. A yearlong measurement that was conducted in the Negev has found that a substantial amount was contributed following vapor emitted from the soil. This vapor, termed indirect rainfall water (IRW), was found to provide an average daily amount of 0.12 mm and 0.07 mm of water to the cobbles and rock slabs, respectively (Kidron and Kronenfeld 2023).

Hypothesizing that along with direct rain and NRW, IRW, whether in the form of vapor or liquid water (distillation), may also serve as an important water source for lithic organisms, assessment of the amount and duration of these water sources for the lithobiont community was called for. Our measurements were conducted in the Negev Highlands during three years (beginning of September 2021 to the end of August 2024).

## Materials and methods

### The research site

**Fig. 1:**
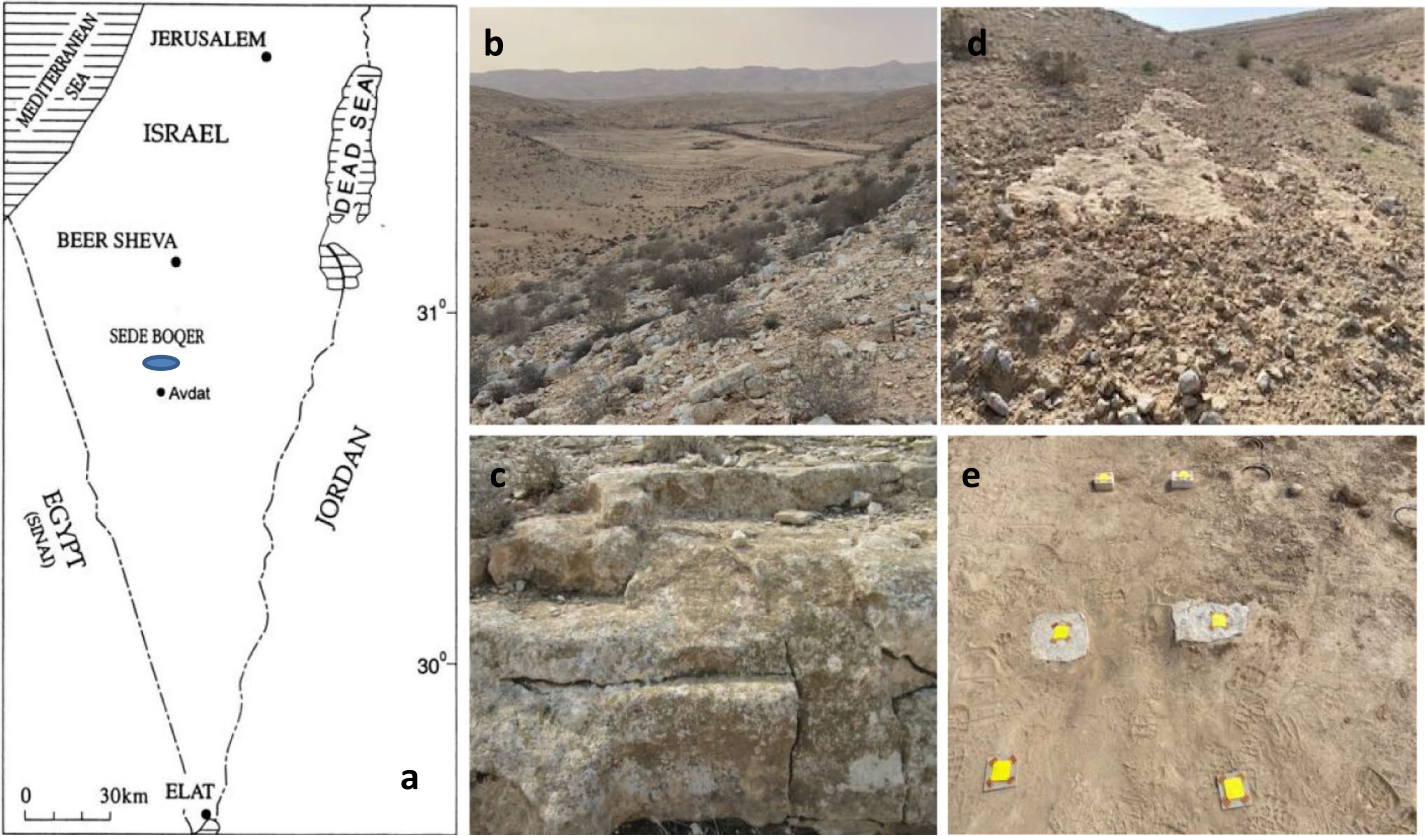
Location of the research site (**a**), general view of the hills bordering the Zin Valley (**b**), chlorolichens covering the north-facing bedrocks (**c**), isolated rock outcrops surrounded by stones and cobbles (**d**), and NRW measurements on cobbles (top), rock slabs (middle), and the CPM (bottom)

The research was conducted in the Sede Boqer Research Site (SBRS) in the heart of the Negev Highlands (34°23′E, 30°56′N). It is located in a 7 km × 3 km loessial valley (Sede Zin) at 480 m above msl. SBRS lies near Kibbutz Sede Boqer, adjacent (150 m) to a standard meteorological station run by the Israel Meteorological Service, IMS (Fig. 1a),

Long-term precipitation is 95 mm, falling between November and April (Rosenan and Gilad 1985). Dew (and distillation) is frequent, forming during ~ 200 days a year, providing ~ 33 mm of water a year (Evenari et al. 1971). Average annual temperature is 17.9 °C; average daily temperature is 24.7 °C during the warmest month of July and 9.3 °C during the coldest month of January (Bitan and Rubin 1991). Annual potential evaporation is ~ 2600 mm (Evenari 1981).

Sede Zin borders low (50–70 m) Turonian limestone hills sparsely covered (5–15%) by dwarf shrubs (commonly < 40 cm high). Whereas *Artemisia herba-alba* Asso. and *Gymnocarpos decandrum* Forssk. characterize the shaded north- and west-facing slopes, *Zygophyllum dumosum* Boiss. and *Haloxylon scoparium* Pomel characterize the sun-exposed south- and east-facing slopes, with *Retama raetam* (Forssk.) Webb and *H. scoparium* characterizing the wadis. The rocky slopes are characterized by continuous bedrock at the midslopes and rocky terraces with alternating rock ledges and soil strips at the upper and lower slope sections (Fig. 1b and c). The slopes are covered by numerous stones and cobbles, having occasionally isolated rock outcrops (Fig. 1d). A lush population of lithobionts (> 90%) covers the rock surfaces, both chlorolichens and cyanobacteria. While cyanobacteria are confined to the midslopes of the sun-exposed massive south-facing (Kidron et al. 2014) and east-facing (Kidron et al. 2016) bedrocks (with *Gloeocapsa* sp. predominating), chlorolichens cover all other rock surfaces, as well as all cobbles, regardless of aspect (Kidron et al. 2011).

While endolithic cyanobacteria (*Gloeocapsa* sp.) inhabit the bedrocks of the south- and east-facing slopes (Friedmann and Galun 1974), endolithic and especially epilithic lichens, both with green algae as photobionts, i.e., chlorolichens, inhabit the bedrocks of the north- and west-facing slopes. The lichen *Pyrenodesmia alociza* (A. Mass.) Arnold (= *Caloplaca* alociza) predominates among the endolithic lichens, and the predominating epilithic lichens are *Variospora aurantia* (Pers.) Arup, Frödén & Søchting *(*= *Caloplaca aurantia*), *Caloplaca circumalbata* (Delile) Wunder var. *circumalbata* (= *Caloplaca aegyptiaca*), *C. circumalbata* var. *bicolo*r (Müll. Arg.) Wunder, *Diplotomma epipolium* (Ach.) Arnold (= *Buellia epipolia*), *Lobothallia farinosa* (Flörke) A. Nordin, Savić & Tibell (= *Lecanora farinosa*), *Myriolecis albescens* (Hoffm.) Śliwa, Zhao Xin & Lumbsch (= *Lecanora albescens*), and *Tephromela atra* (Huds.) Hafellner (= *Lecanora atra*) (Lange et al. 1970; Friedmann and Galun 1974).

As verified during numerous measurements, while the chlorolichens benefit from dew, this was not the case with cyanobacteria. The rock-dwelling cyanobacteria at the south- and east-facing midslopes never reached the dewpoint temperature, not even reaching the 0.05 mm threshold required for the performance of net photosynthesis by the chlorolichens (Kidron et al. 2014, 2016). The additional amount of water obtained by the lichen-inhabiting cobbles was also supported by the chlorophyll content of the cobbles that was substantially higher than that of the cyanobacteria-inhabiting rock surfaces, 47.3 mg m^2^ and 16.4 mg m^2^, respectively (Kidron et al. 2016).

The above-mentioned measurements were confined to the rainless months and therefore to NRW. They did not include possible wetting by IRW. IRW may result in distillation, but also in high vapor content of ≥ 0.05 mm, which may be sufficiently high to perform net photosynthesis by chlorolichens (Kidron and Kronenfeld 2023). To evaluate the contribution of NRW and IRW, the water content that accumulated on two pairs of 40–60 cm × 30 cm × 5 cm rock slabs (protruding ~ 2 cm above surface) and 10 cm × 10 cm × 5 cm cobbles (lying on the ground) was measured. Whereas cobbles abound on the rocky hill slopes of the Negev Highlands, the rocky surfaces are commonly part of the massive or jointed bedrocks. However, inhabited by cyanobacteria and chlorolichens, isolated rock outcrops that are surrounded by soil can also be found, represented hereafter by the rock slabs.

### Methodology

The measurements took place using 6 cm × 6 cm × 0.1 cm velvet-like PVA microfiber porous cloths (Vileda, Weinheim, Germany) that were attached at their four corners to the cobble and rock surfaces via adhesive tape. For a comparison with past measurements, we also measured the amount of water using the cloth-plate method (CPM) (Kidron 1998). It consists of 6 cm × 6 cm × 0.1 cm cloth attached to a 10 cm × 10 cm × 0.2 cm glass plate, which is glued in turn to a 10 cm × 10 cm × 0.5 cm plywood plate, thus creating an identical substrate, 0.7 cm above ground (Fig. 1e).

The cloths were attached to each of the substrates each afternoon (~ 16:00) and collected during the early morning hours (around sunrise). Each cloth was put into a glass flask that was immediately sealed. The flasks were taken to a nearby laboratory where they were weighed, oven-dried (70 °C until reaching a constant weight) and then re-weighed. The amount of water (whether as vapor or liquid) was calculated according to the equation:

1 $${\mathrm{WC}}\left( {{\mathrm{mm}}} \right) = \left( {\left( {{\mathrm{WC}}_{{{\mathrm{wet}}}} {-}{\mathrm{WC}}_{{{\mathrm{dry}}}} } \right)/A\rho } \right) \times {1}0$$

where WC is the amount of water in millimeters, WC_wet_ and WC_dry_ are the wet and dry water contents of the cloths in grams, respectively; *A* is the surface area in cm^2^, and *ρ* is the density of water (g cm^−3^) at a given temperature, multiplied by 10 to convert the values to millimeters.

With vapor originating from the soil as long as the upper 3 cm of the soil remains wet (i.e., above the wilting point), we regarded the conditions as wet conditions and the accumulated water as IRW (Kidron et al. 2022). This was reflected once condensation was noted to cover > 50% of the soil-facing bottom of a pair of 10 cm × 10 cm × 0.2 cm glass plates located on soil. Water that was considered to result from IRW was therefore confined to days that followed a rain event. As long as the upper 3 cm of the soil remained dry, it was considered to reflect dry conditions (regardless of season), and the accumulated water was attributed to NRW. Once condensation takes place, it is termed dew when the vapor stems from the atmosphere or distillation when stemming from the soil. We are aware of the possible occurrence of both processes concomitantly, as verified by the seminal paper published by Kaseke et al. (2017) in which the differentiation between dew and distillation was based on isotopic analysis. However, focusing at the near surface, which is largely impacted by the soil vapor and the fact that following rain the required temperature gradient that facilitates the sea breeze (which carries vapor from the Mediterranean Sea to the desert) is unlikely (Bitan and Rubin 1991), we regarded the accumulated vapor during days that follow rain events as IRW. We also maintain that while a small amount of NRW cannot be excluded during these wet conditions, a small amount of IRW cannot be excluded even when the upper 3 cm of the soil dries out, as some vapor may still be emitted from the soil, as evidenced by vapor condensation at the bottom of the glass plate. We believe that these processes may cancel each other out and the distinction between NRW and IRW is generally sound. Therefore, similarly to attempts to differentiate between two types of fog (Kaseke and Wang 2022), a differentiation between NRW and IRW may be feasible.

As for fog, it was determined visually, once visibility was restricted to < 1 km (Fairbridge 1967). We are aware of the fact that our visual observation noted the occurrence of fog only when the fog lasted until the morning. However, we assume that (1) due to the likelihood of fog to form during the early morning (when temperatures are minimal), we visually noted almost all fogs, and (2) due to the similar accumulation pattern and the common co-occurrence of dew and fog in the Negev (Levi 1967; Kidron et al. 2000), the amounts and pattern obtained are reliable.

With microorganism growth being principally determined by available water duration rather than by water amount (Kappen et al. 1980; Kidron et al. 2011), the relationship between the amount of NRW and its duration (as found for the CPM in the Negev) served to assess the potential water duration, in accordance with Eq. (2) (Kidron et al. 2000):

2 $${\mathrm{NRW}}_{{{\mathrm{dur}}}} = {1}.{147}\;{\mathrm{ln}}\left( {{\mathrm{NRW}}_{{\mathrm{q}}} } \right) + {5}.{21}$$

where NRW_dur_ is the duration of NRW (h) and NRW_q_ is the quantity (mm).

One-way ANOVA with Bonferroni as a post-hoc test was executed (using SPSS 11.0 for Windows; SPSS, Chicago, IL) in order to examine possible significant differences between the amount of NRW or IRW as obtained by the cobbles or rock slabs. Once significant, paired *t*-tests were performed. Differences were regarded as significant at *P* < 0.05.

## Results

**Fig. 2:**
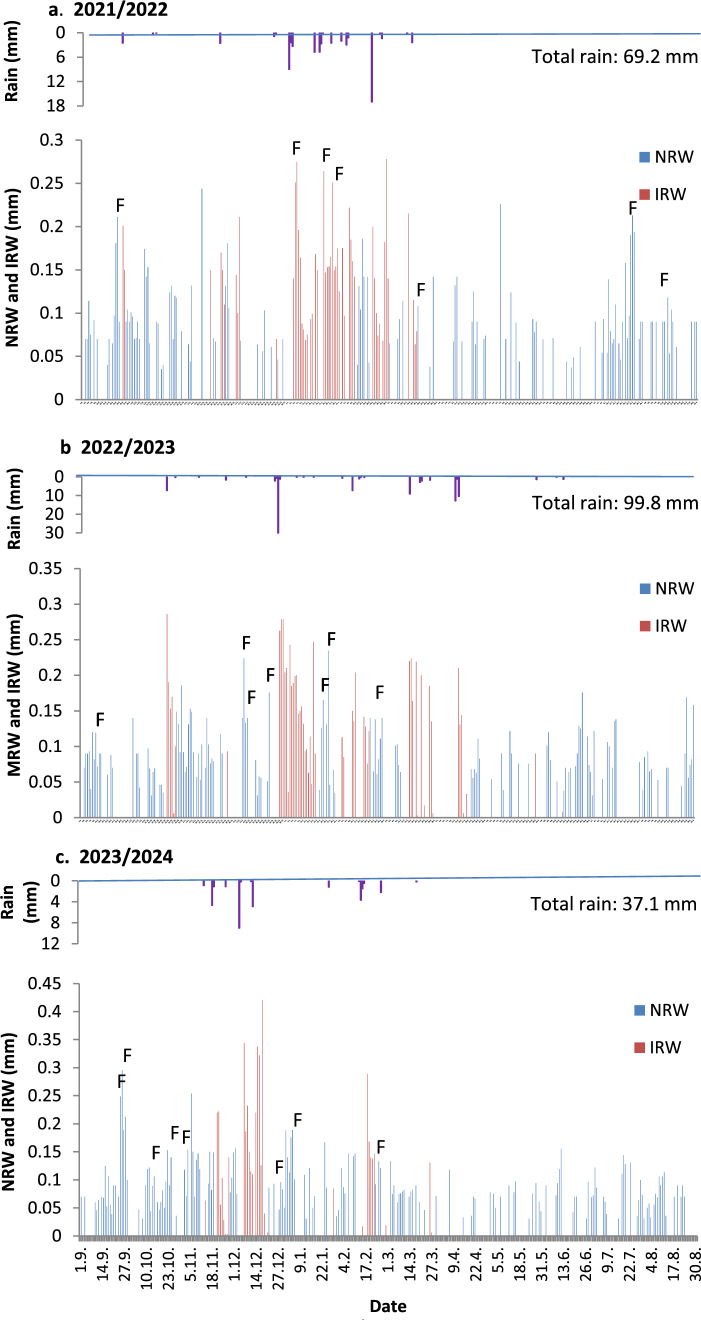
Daily distribution of rain, NRW and IRW. The letter F denotes fog

Rain precipitation during the research period was 69.2, 99.8, and 37.1 mm for 2021/22, 2022/23, and 2023/24, respectively, yielding an average of 68.7 mm. The amounts were lower than the 95 mm long-term mean, with 2023/24 yielding a particularly low amount, reflecting a severe drought. The distributions of rain, NRW, and IRW are shown in Fig. 2. Whereas NRW took place during the entire year, IRW was confined to a limited number of winter days following rain events. As for fog, it was not common. Fog was recorded for only 28 days (25 and 3 days during the dry and wet periods, respectively), i.e., 9 (SD = 2.5) days per year, i.e., 5.0% of all days that yielded vapor accumulation. Since fog commonly occurs concomitantly with dew, dew and fog were jointly analyzed as NRW and IRW during the dry and wet conditions, respectively.

**Fig. 3:**
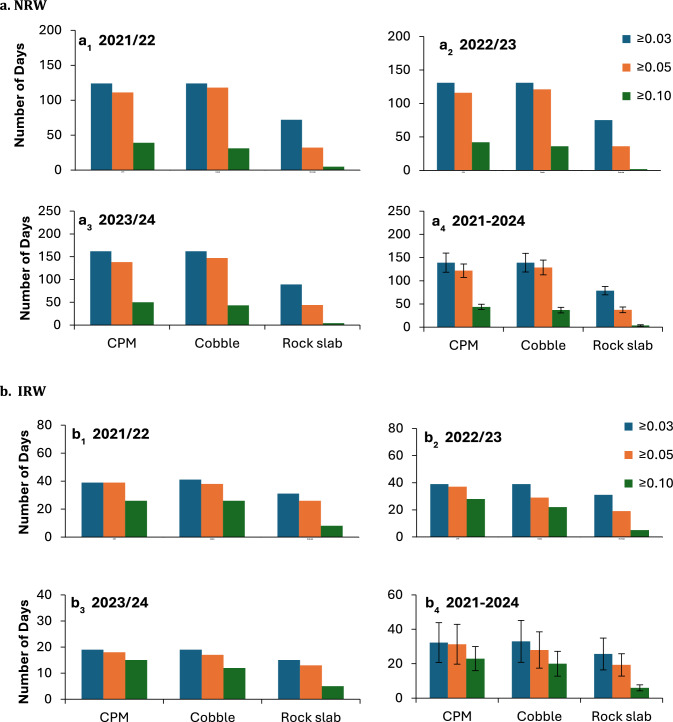
The number of days with NRW (**a**) and IRW (**b**) with ≥ 0.03, ≥ 0.05, and ≥ 0.1 mm as measured by the CPM, and on cobbles and rock slabs during 2021/22 (**a**_**1**_, **b**_**1**_), 2022/23 (**a**_**2**_, **b**_**2**_), 2023/24 (**a**_**3**_, **b**_**3**_), and the average number of days during 2021–2024 (**a**_**4**_, **b**_**4**_). Bars represent one standard deviation

The number of days with ≥ 0.03 mm, ≥ 0.05 mm and ≥ 0.1 mm for NRW are shown in Fig. 3a, while the number of days with ≥ 0.03 mm, ≥ 0.05 mm and ≥ 0.1 mm for IRW are shown in Fig. 3b. Extra attention was given not include drizzle within the IRW days.The number of days with ≥ 0.03 mm and ≥ 0.1 mm was highest and lowest, respectively. While the number of days recorded by the CPM and the cobbles was similar, the number recorded by the rock slabs was lower, especially for NRW, a point that will be dealt with below. A low number of days with IRW also characterizes 2023/2024, reflecting the extremely small amount of rain that fell during this year.

**Fig. 4:**
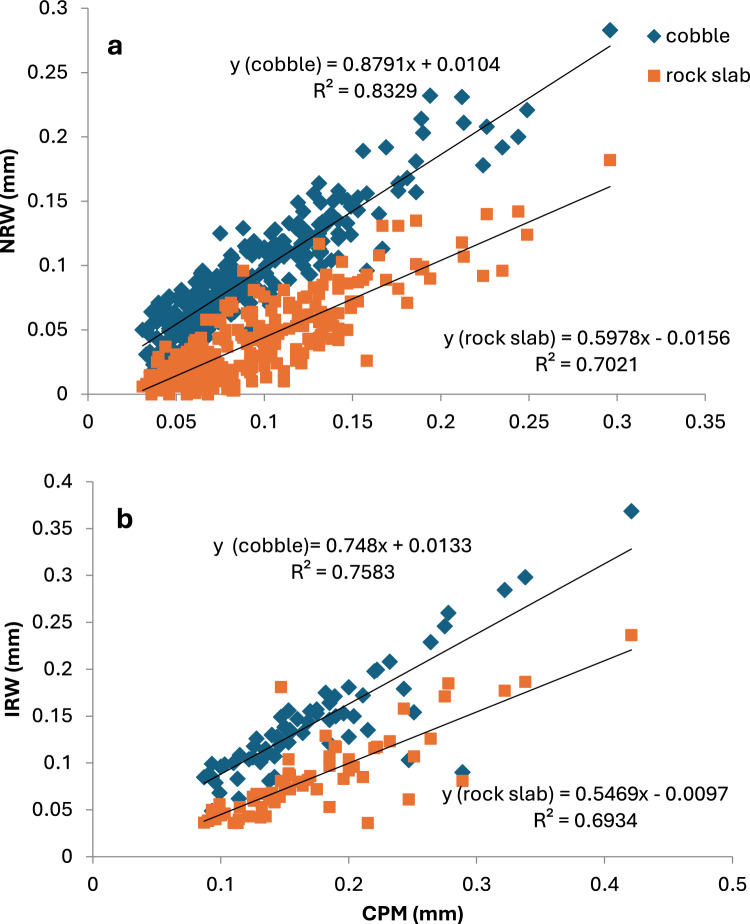
The relationships between CPM and NRW (**a**) and IRW (**b**) as received on cobbles and isolated rock outcrops

The daily amounts of NRW and IRW show substantial differences in the daily amounts of NRW and IRW (Fig. 4a, b and Table 1a, b)**.** All NRW and IRW values exhibited significant differences between the substrates, with CPM > cobbles > rock slabs. As for dew and distillation, the average daily amount of dew for the CPM and the cobbles was 0.14 mm and 0.13 mm, respectively, while being 0.12 mm for the rock slabs. Similarly, the average amount of distillation for the CPM and cobbles was 0.18 mm and 0.16 mm, respectively; it was 0.14 mm for the rock slabs. As for the annual amounts, the data show a higher similarity between the amounts obtained by the CPM and the cobbles in comparison to the rock slabs. Thus, in comparison to the annual amounts of dew and distillation of 0.5 mm and 0.9 mm received by the rock slabs, respectively, 4.7 mm and 3.1 mm of distillation were received by the cobbles, respectively (Table 2).

**Table 1:** Daily amount of NRW (**a**) and IRW (**b**) as obtained on CPM, Cobbles and rock slabs

(a)									
Year	Daily amount of NRW (mm)								
	CPM			Cobble			Rock		
	≥ 0.03	≥ 0.05	≥ 0.10	≥ 0.03	≥ 0.05	≥ 0.10	≥ 0.03	≥ 0.05	≥ 0.10
2021/22	0.09 (0.04)	0.10 (0.04)	0.14 (0.04)	0.09 (0.03)	0.09 (0.03)	0.13 (0.03)	0.06 (0.02)	0.08 (0.02)	0.11 (0.01)
2022/23	0.09 (0.04)	0.10 (0.04)	0.14 (0.03)	0.09 (0.03)	0.09 (0.03)	0.12 (0.02)	0.05 (0.02)	0.07 (0.02)	0.12 (0)
2023/24	0.09 (0.04)	0.10 (0.04)	0.14 (0.04)	0.09 (0.03)	0.09 (0.03)	0.13 (0.03)	0.06 (0.02)	0.07 (0.02)	0.14 (0.02)
Average (SD)	0.09 (0.02)	0.10 (0.00)	0.14 (0.00)	0.09 (0.00)	0.09 (000)	0.13 (0.00)	0.05 (0.00)	0.07 (0.00)	0.12 (0.01)

(b)									
Year	Daily amount of IRW (mm)								
	CPM			Cobble			Rock		
	≥ 0.03	≥ 0.05	≥ 0.10	≥ 0.03	≥ 0.05	≥ 0.10	≥ 0.03	≥ 0.05	≥ 0.10
2021/22	0.15 (0.06)	0.15 (0.06)	0.13 (0.05)	0.12 (0.05)	0.13 (0.05)	0.15 (0.04)	0.08 (0.04)	0.09 (0.04)	0.14 (0.03)
2022/23	0.14 (0.04)	0.14 (0.04)	0.16 (0.04)	0.11 (0.04)	0.11 (0.04)	0.14 (0.03)	0.07 (0.03)	0.09 (0.03)	0.12 (0.02)
2023/24	0.12 (0.10)	0.18 (0.10)	0.20 (0.10)	0.14 (0.09)	0.16 (009)	0.19 (0.09)	0.10 (0.06)	0.11 (0.06)	0.17 (0.05)
Average (SD)	0.15 (0.02)	0.15 (0.02)	0.18 (0.02)	0.12 (0.02)	0.13 (0.02)	0.16 (0.03)	0.08 (0.02)	0.10 (0.01)	0.14 (0.02)

**Table 2:** Annual amount of water as received for the CPM, cobbles and rock slabs as a result of NRW (**a**) and IRW (**b**) for days with ≥ 0.03, ≥ 0.05, and ≥ 0.1 mm

(a)									
Year	Annual amount of NRW (mm)								
	CPM			Cobble			Rock		
	≥ 0.03	≥ 0.05	≥ 0.10	≥ 0.03	≥ 0.05	≥ 0.10	≥ 0.03	≥ 0.05	≥ 0.10
2021/22	11.7	11.2	5.6	10.9	10.7	4.1	4.0	2.4	0.6
2022/23	11.9	11.3	5.8	11.2	10.7	4.4	4.0	2.5	0.2
2023/24	14.8	13.9	7.2	13.9	13.2	5.5	5.0	3.2	0.5
Average (SD)	12.8 (1.7)	12.1 (1.5)	6.2 (0.9)	12.0 (1.6)	11.5 (1.5)	4.7 (0.8)	4.3 (0.6)	2.7 (0.5)	0.5 (0.2)

(b)									
Year	Annual amount of IRW (mm)								
	CPM			Cobble			Rock		
	≥ 0.03	≥ 0.05	≥ 0.10	≥ 0.03	≥ 0.05	≥ 0.10	≥ 0.03	≥ 0.05	≥ 0.10
2021/22	5.7	5.7	4.7	4.8	4.8	3.9	2.6	2.4	1.1
2022/23	5.3	5.2	4.5	4.3	4.2	3.0	2.1	1.6	0.5
2023/24	3.3	3.2	3.0	2.7	2.6	2.2	1.5	1.4	0.8
Average (SD)	4.8 (1.3)	4.7 (1.3)	4.1 (0.9)	4.0 (1.1)	3.9 (1.1)	3.1 (0.8)	2.1 (0.6)	1.8 (0.5)	0.9 (0.2)

As far as the daytime duration is concerned, the daytime duration of NRW of the CPM and the cobbles were similar (2.3–2.9 h); also, the daily amounts of IRW for the CPM and cobbles (2.8–3.2 h). The rock slabs yielded, however, substantially shorter durations for NRW (1.8–2.8 h) and IRW (2.2–2.9 h) (Table 3). Subsequently, the annual durations of NRW and IRW of the cobbles (and CPM) were substantially longer than those for the rock slabs, with NRW yielding substantially longer durations than IRW (Table 4). Annual daytime duration following NRW was the longest, with amounts of ≥ 0.03 and ≥ 0.05 lasting for 325.0 and 307.8 h for the cobbles and 141.7 and 81.0 h for the rock slabs, respectively. Dew (amounts ≥ 0.1 mm) yielded substantially shorter durations, 103.8 h and 10.3 h for cobbles and rock slabs, respectively. As for IRW, the daytime duration following IRW was substantially shorter, 87.1 and 56.4 h during days with ≥ 0.03 and 0.05 mm for the cobbles and the rock slabs, respectively, while the annual duration during which daily amounts of ≥ 0.1 mm occurred was 60.2 h (for cobbles) and 17.6 h (for rock slabs). The findings highlight the relatively short duration of dew relative to NRW at the rock slabs (7.3%) in comparison to the cobbles (31.9%). On the other hand, the annual daytime duration during which distillation occurred in comparison to IRW was relatively long for rock slabs (31.2%) and especially long for the cobbles (69.1%).

**Table 3:** Daily duration of NRW (**a**) and IRW (**b**) as obtained on CPM, cobbles and rock slabs

(a)									
Year	Daily duration of NRW (mm)								
	CPM			Cobble			Rock		
	≥ 0.03	≥ 0.05	≥ 0.10	≥ 0.03	≥ 0.05	≥ 0.10	≥ 0.03	≥ 0.05	≥ 0.10
2021/22	2.40 (0.48)	2.50 (0.43)	2.95 (0.28)	2.37 (0.36)	2.40 (034)	2.86 (0.21)	1.81 (0.42)	2.21 (0.31)	2.72 (0.11)
2022/23	2.35 (0.49)	2.47 (0.40)	2.91 (0.23)	2.33 (0.53)	2.38 (0.32)	2.80 (0.17)	1.78 (0.40)	2.13 (0.25)	2.79 (0.04)
2023/24	2.34 (0.54)	2.49 (0.43)	2.95 (0.23)	2.32 (0.40)	2.69 (0.35)	2.83 (0.22)	1.81 (0.43)	2.17 (0.31)	2.91 (0.18)
Average (SD)	2.36 (0.04)	2.49 (0.02)	2.94 (0.02)	2.34 (0.02)	2.39 (0.01)	2.83 (0.03)	1.80 (0.02)	2.17 (0.04)	2.81 (0.10)

(b)									
Year	Daily duration of IRW (mm)								
	CPM			Cobble			Rock		
	≥ 0.03	≥ 0.05	≥ 0.10	≥ 0.03	≥ 0.05	≥ 0.10	≥ 0.03	≥ 0.05	≥ 0.10
2021/22	2.91 (0.05)	2.91 (0.49)	3.20 (0.28)	2.82 (0.62)	2.73 (0.43)	3.00 (0.27)	2.25 (0.53)	2.42 (0.40)	2.91 (0.28)
2022/23	2.81 (0.52)	2.88 (0.43)	3.07 (0.30)	2.59 (0.48)	2.65 (0.40)	2.92 (0.22)	2.04 (0.48)	2.34 (0.35)	2.79 (0.18)
2023/24	2.99 (0.22)	3.08 (0.65)	3.27 (0.51)	2.78 (0.70)	2.92 (0.61)	3.19 (0.49)	2.40 (0.60)	2.52 (0.55)	3.12 (0.34)
Average (SD)	2.91 (0.09)	2.96 (0.10)	3.18 (0.10)	2.95 (0.11)	2.77 (0.14)	3.04 (0.14)	2.23 (0.18)	2.43 (0.09)	2.94 (0.17)

**Table 4:** Annual wetness duration (h) for the CPM, cobbles and rock slabs as a result of NRW (**a**) and rain (**b**) and IRW for days with ≥ 0.03, ≥ 0.05 and ≥ 0.1 mm

(a)									
Year	Annual duration of NRW (h)								
	CPM			Cobble			Rock		
	≥ 0.03	≥ 0.05	≥ 0.10	≥ 0.03	≥ 0.05	≥ 0.10	≥ 0.03	≥ 0.05	≥ 0.10
2021/22	298.1	277.7	115.1	293.4	283.1	88.7	130.7	70.7	13.6
2022/23	308.1	286.0	122.3	305.1	288.4	100.7	133.1	76.8	5.6
2023/24	378.8	344.0	147.7	376.3	351.8	121.9	161.3	95.7	11.6
Average (SD)	328.3 (94.0)	302.5 (36.1)	128.3 (17.1)	325.0 (44.9)	307.8 (78.2)	103.8 (16.8)	141.7 (17.0)	81.0 (13.0)	10.3 (4.3)

(b)										
Year	Annual duration of rain and IRW (h)									
	Rain	IRW								
	CPM/Cob/Rock	CPM			Cobble			Rock		
		≥ 0.03	≥ 0.05	≥ 0.1	≥ 0.03	≥ 0.05	≥ 0.1	≥ 0.03	≥ 0.05	≥ 0.1
2021/22	114.0	113.5	113.5	83.3	107.4	103.4	78.1	69.8	61.8	23.3
2022/23	138.0	109.8	106.7	85.8	101.0	98.1	64.1	63.2	44.5	14.0
2023/24	59.0	56.9	55.4	49.0	52.9	49.6	38.2	36.0	32.8	15.6
Average	103.7 (40.5)	93.4 (31.7)	91.9 (31.8)	72.7 (20.6)	87.1 (29.8)	83.9 (17.7)	60.2 (20.2)	56.4 (17.9)	46.7 (15.4)	17.6 (5.0)

With regards to the amounts provided by dew and distillation in comparison to rain, dew and distillation provided only 7.8 mm (11.4%) and 1.4 mm (2.0%) to the cobbles and rock slabs, respectively. Nevertheless, in comparison to the surface wetness duration of rain of 103.7 h, dew and distillation provided together 164.0 h and 27.9 h for the cobbles and rock slabs, respectively, with distillation providing 36.7% and 63.1% of the vapor-driven liquid for the cobbles and rock slabs, respectively.

## Discussion

The lush cover of lithobionts in the Negev Desert is rather unique. The authors are not aware of other hyper-arid deserts with > 80–90% cover of crustose lithic lichens. These unique ‘lichen forests’ were attributed to the high frequency of dew, given the proximity of the Negev Desert and especially the Negev Highlands to the Mediterranean Sea. This guarantees a continuous source of vapor, which, coupled with adiabatic cooling, is seen as responsible for the high occurrence of dew at the Negev Highlands at 300–1000 m amsl, and subsequently for the high cover of lichens on the rocky outcrops and cobbles (Kidron and Temina 2013).

Recent research highlighted the occurrence of WDCs in the Negev (Kidron et al. 2022, 2025) and subsequently the potentiality of vapor that stems from the soil to provide sufficient vapor to above-surface substrates such as rock slabs and cobbles (Kidron and Kronenfeld 2023). The findings implied that in addition to NRW, whether dew, fog, or high RH, IRW may also provide water to lithobionts. However, as substantiated following previous research (Kidron and Kronenfeld 2023), the effect may differ for rock outcrops and cobbles. As previously reported, NRW measured on rock outcrops within a small drainage basin showed high variability (Kidron et al. 2000). While most outcrops benefit from NRW, massive sun-exposed rock outcrops at the south- and east-facing slopes failed to yield ≥ 0.05 mm of vapor required for the performance of net photosynthesis by lichens. Amounts of ≥ 0.05 mm and even ≥ 0.1 mm were, however, received on rock surfaces at the remaining (cooler) aspects and on all cobbles, regarding of aspect, which were covered indeed by lichens. The differences in NRW were explained by differences in surface temperatures. High incoming radiation and efficient heat storage of the sun-exposed rock outcrops resulted in slow nocturnal radiative cooling, which was reflected by the high nocturnal temperatures at these rock surfaces. These relatively high nocturnal temperatures did not facilitate vapor condensation, nor sufficiently high RH that may facilitate NRW amounts of ≥ 0.05 mm (Kidron et al. 2014, 2016). Similarly, radiative cooling was also impeded by partially buried cobbles, resulting in about half the amount of NRW in comparison to detached (loose) cobbles of similar dimensions (Kidron 2000b).

The NRW amounts that accumulated on the cobbles were substantially higher than those accumulating on the rock slabs. They were similar (although slightly lower) to the values obtained by the CPM. The slightly higher amounts obtained by the CPM can be explained by the thermal properties of the glass that yielded about 15% higher NRW in comparison to an identical size glass plate (Kidron 1998).

In comparison to the rock slabs, higher nocturnal temperatures characterized the cobble. The cobbles also accumulated higher amounts of IRW relative to the rock slabs. Since temperature-induced vapor flux, which is reflected by WDCs, is confined to porous substrates and therefore takes place in soils, IRW may therefore affect rock substrates surrounded by soil, whether cobbles or isolated rock outcrops. Having only limited soil patches, massive rock outcrops are expected, however, to be less affected by IRW. Nevertheless, in comparison to the tenfold higher annual amount of dew that was condensed on the cobbles in comparison to the rock slabs (4.7 mm in comparison to 0.5 mm; Table 2), the amount of distillation that accumulated on the rock slabs was relatively high. The relatively high amount of distillation recorded on the rock slabs reflects the cardinal effect of the surrounding wet soil on the rock slabs, explained by the proximity to the vapor source. Unlike massive bedrocks that are commonly not surrounded by soil, isolated rock outcrops, which are reflected herein by the rock slabs, may therefore benefit from additional water through IRW.

With surface wetness duration mainly dictating lithobiont growth (Kappen et al. 1980; Kidron et al. 2011), the findings highlight the role played by NRW, especially for cobbles. Concerning the cobbles, dew provided an equivalent duration (103.8 h) to rain (103.7 h), while distillation provided an additional duration of 60.2 h. As far as the rock slabs are concerned, rain was the main source of water (103.7 h) in comparison to only 10.3 h of dew and 17.6 h of distillation, explained by the higher nocturnal surface temperatures of the partially buried rock slabs in comparison to the loose cobbles (Kidron 2000b; Kidron and Kronenfeld 2023).

The lower nocturnal temperatures of the cobbles in comparison to the rock slabs (by 0.8 ℃; Kidron and Kronenfeld 2023) may explain the higher amount and subsequently the longer surface wetness duration of the cobbles. This may also explain the high chlorophyll content that characterizes the cobbles in comparison to the rock surfaces (Kidron et al. 2014), highlighting the great effect that minute temperature differences may have on non-vascular organisms (Stanton et al. 2014). With massive bedrocks being characterized by higher nocturnal temperatures than the rock slabs and following the fact that these rock outcrops are commonly not surrounded by soil, IRW may only marginally affect massive rock outcrops. Since sun-facing massive rock outcrops do not also benefit from NRW > 0.05 mm, these outcrops will only benefit from rainwater. This may explain the ~ threefold lower chlorophyll content of these outcrops (Kidron et al. 2014) in comparison to the adjacent cobbles, which also benefit from NRW and IRW.

As far as the hydrology is concerned, the current findings point to a clear distinction that should be made between NRW and IRW. Not all vapor can be regarded as NRW and not all the liquid water that stems from vapor condensation can be regarded as dew. Since some of the condensed vapor is distillation, it implies that this water reflects water redistribution, and therefore, cannot be regarded as water addition to the ecosystem. The findings suggest that IRW may provide water to lithobionts also in non-dewy deserts, such as the deserts in the southwestern parts of the USA. Indeed, chlorolichens inhabit rock surfaces also of non-dewy deserts such as the Mojave (Nash et al. 1977; Nash and Moser 1982; Bull 2004; Cung et al. 2021), the Chihuahuan Desert (Nash et al. 1977; Nash and Moser 1982), and the interior parts of the Sonoran Desert (Nash et al. 1977; Nash and Moser 1982; Garvie et al. 2008). No dew or fog was detected in the Mojave based on a careful microclimatological analysis (Nash et al. 1977), and the authors concluded that rain serves as the only water source. However, Nash et al. (1977) also commented that the only days during which potential vapor condensation was feasible were following winter rain events during which high RH was recorded. Based on our current data, this may reflect the occurrence of WDCs and subsequently of IRW and distillation, as also reported from the Negev (Kidron et al. 2022). As outlined above, distillation not only wets the soil surface but may also result in vapor condensation on the rock surfaces, especially at lower heights above ground and on rock fragments, whether boulders or cobbles (Bull 2004; Cung et al. 2021). This was also noted in the Chihuahuan Desert where the bottom parts of rocks were covered by lichens (personal observations by GJK in the vicinity of Las Cruces, New Mexico).

IRW may thus play an important ecological role. Whereas the time duration during which direct rain wets the rocky substrates is limited, IRW grants additional activity time to lithic chlorolichens, whether on cobbles or isolated rock outcrops. This distinction between NRW and IRW should also be taken into account in model calculations and when evaluating the possible effect of global warming and climate change.

Regarding the climate change, the current findings may have important implications. Climate change may result in increased droughts (Dai 2013) and increased nocturnal warming (Price et al. 1999; Morak et al. 2011; Donat and Alexander 2012), each of which may pose a particular effect on the lithobiont population. A change in the rain regime and global warming may differentially affect NRW and IRW. Thus, NRW will be specifically affected by global warming that may increase the nighttime temperatures and will impede both dew and distillation. A reduction in rain precipitation will reduce the number and magnitude of the WDCs and may negatively affect IRW. A combination of both phenomena (temperature increase and rain reduction) may have an especially hazardous effect on lithobiont growth and distribution.

## Conclusions

The amounts and duration of non-rainfall water (NRW) and indirect rainfall water (IRW) that were absorbed by cloths attached to cobbles and rock slabs and recorded by the CPM were quantified during three years of measurements in the Negev Highlands. Several conclusions emerged. (1) Both phenomena, NRW and IRW, should be dealt with separately. The annual amount of NRW and IRW was substantially higher for cobbles, explained by the efficient nocturnal heat loss by these loose rock particles. While the effect of IRW upon massive bedrocks may be limited (due to the limited extent of the surrounding soil), IRW may have a relatively high effect on isolated rock outcrops, represented herein by the rock slabs. (2) In comparison to rocks, cobbles not only benefit from higher amounts of NRW but also from higher amounts of IRW, and thus, although being confined to limited days after rain events, IRW provided a meaningful daytime wetness duration, especially to cobbles, and may therefore be regarded as an important water source for lithobiont-inhabiting cobbles and isolated rock outcrops. (3) IRW may thus provide water also to non-dewy deserts. These findings should be considered in future models aiming to study the possible effects of climate change on lithobionts.

## Data Availability

Data will be made available on reasonable request.

## Abbreviations

CPM: Cloth–plate method
IRW: Indirect rain water
NRW: Non-rainfall water
RH: Relative humidity
WDC: Wet–dry cycles
